# Revealing defect-bound excitons in WS_2_ monolayer at room temperature by exploiting the transverse electric polarized wave supported by a Si_3_N_4_/Ag heterostructure

**DOI:** 10.1515/nanoph-2023-0560

**Published:** 2023-11-22

**Authors:** Shulei Li, Fu Deng, Lidan Zhou, Zhenxu Lin, Mingcheng Panmai, Shimei Liu, Yuheng Mao, Jinshan Luo, Jin Xiang, Jun Dai, Yunbao Zheng, Sheng Lan

**Affiliations:** School of Optoelectronic Engineering, Guangdong Polytechnic Normal University, Guangzhou 510665, China; Department of Physics, Hong Kong University of Science and Technology, Kowloon, Hong Kong, China; State Key Laboratory of Optoelectronic Materials and Technologies and School of Electronics and Information Technology, Sun Yat-Sen University, Guangzhou 51006, China; Guangdong Provincial Key Laboratory of Nanophotonic Functional Materials and Devices, School of Information and Optoelectronic Science and Engineering, South China Normal University, Guangzhou 510006, China; Key Laboratory of Optoelectronic Technology and Systems (Chongqing University), Ministry of Education, School of Optoelectronic Engineering, Chongqing University, Chongqing 400044, China

**Keywords:** monolayer transition-metal dichalcogenides, dielectric-metal heterostructure, transverse-electric-polarized wave, defect-bound exciton, photoluminescence patterns

## Abstract

Two-dimensional (2D) transition metal dichalcogenide (TMDC) monolayers are promising materials for light-emitting devices due to their excellent electric and optical properties. However, defects are inevitably introduced in the fabrication of TMDC monolayers, significantly influencing their emission properties. Although photoluminescence (PL) is considered as an effective tool for investigating the defects in TMDC monolayers. However, the PL from the defect-bound excitons is revealed only at low temperatures. Here, we show that the PL from the defect-bound excitons in a WS_2_ monolayer can be effectively revealed at room temperature by exploiting the transverse electric polarized wave supported by a Si_3_N_4_/Ag heterostructure. It is revealed that the defect-bound excitons in all possible positions of the WS_2_ monolayer can be effectively excited by the TE wave with significantly enhanced in-plane electric field localized on the surface of the Si_3_N_4_ layer. In addition, the emission from defect-bound excitons can propagate to the collection point with small attenuation. More importantly, the exciton dynamics in the WS_2_ monolayer can be modified by the Si_3_N_4_/Ag heterostructure, allowing the simultaneous excitation of neutral excitons, charge excitons (trions), and defect-bound excitons in the WS_2_ monolayer attached on the Si_3_N_4_/Ag heterostructure. We inspect the PL spectra obtained at different positions and find that the relative intensity of defect-bound excitons depends on the collection position. We also examine the dependences of the PL intensity and bandwidth on the excitation power for the three types of excitons. It is found that they exhibit different behaviors from those observed in the optical measurements by using the traditional excitation method. Our findings suggest a new way for exciting and studying the dynamics of multi-excitons at room temperature and indicate the potential applications of the TE wave in probing the defects in TMDC monolayers.

## 1 Introduction

Monolayer transition-metal dichalcogenides (TMDCs) have gained enormous research interest because of their unique electronic and optical properties, such as strong nonlinear effect [1–4], complex excitons response and valley selective properties [5–9]. These properties make them important in light–matter interaction and optoelectronic applications [10–19]. Nowadays, monolayer TMDCs with large sizes can be obtained by using the chemical vapor deposition (CVD) method. As a result, hybrid structures composed of metasurfaces and monolayer TMDCs, in which light–matter interaction is greatly enhanced for various applications [20, 21], can be readily fabricated. However, previous studies indicate the existence of various intrinsic/extrinsic defects in monolayer TMDCs grown by the CVD method, such as vacancies, substitutional impurities, grain boundaries, and adatoms [22, 23], based on optically and chemically modulated methods [6, 7, 9, 20, 24, 25] and solution-based liquid-phase exfoliation. These defects exhibit a significant impact on the optical and optoelectronic properties of monolayer TMDCs [23, 26]. While defects are not always detrimental, it is known that point defects in a semiconductor can act as efficient traps to capture free carriers and localize excitons, strongly influencing the transport and optical properties of the host material [27–30]. In particular, such traps become more efficient in low-dimensional materials [26]. Since design and engineering of defects provide a new route for realizing functional properties of monolayer TMDCs, it has become a research hotspot in recent years.

Many gas molecules and chemical substances can effectively functionalize the surface of a two-dimensional material by adjusting defect-induced doping or repairing lattice structure, leading to enhanced luminescence and photo-response [7, 22, 31], [32], [33], [34]. Ion irradiation and plasma treatment are confirmed to be effective methods for creating atomic defects in two-dimensional materials, such as graphene [35], WS_2_ [36], MoS_2_ [37], WSe_2_ [38, 39], ReS_2_ [40], etc. For instance, site-controlled single-photon emitters in monolayer WSe_2_ with high yield was successfully demonstrated [38]. With the rapid progress in the integration of monolayer TMDCs in practical devices, it is highly desirable to understand the physical properties of the defects in monolayer TMDCs and optical characterization appears to be an effective tool to achieve this goal.

Currently, the main techniques used to characterize the defects in monolayer TMDCs include electron beam imaging (e.g., scanning electron microscopy or SEM and transmission electron microscopy or TEM) and fluorescence spectroscopy [22]. Although electron beam imaging provides images at the nanoscale, the introduction of defects is generally inevitable [41]. Therefore, fluorescence characterization, with advantages of high sensitivity, nondestructiveness, and real-time capability, has been widely used [9, 31]. In the photoluminescence (PL) of monolayer TMDCs measured at low temperatures, a new peak was revealed at the long-wavelength side of the neutral excitons. It was attributed to the emission from excitons bound to defects. Unfortunately, the emission from such defect-bound excitons disappears in the PL spectra of monolayer TMDCs at room temperature, making it difficult to study their physical properties.

Physically, the disappearance of the emission from defect-bound excitons is caused by the activation of trapped excitons at room temperature. In order to reveal the emission from defect-bound excitons at room temperature, one needs to enhance the radiative recombination rate of defect-bound excitons, which can be realized by the so-called Purcell effect [42, 43]. In practice, metasurfaces with large field enhancements have been employed to enhance the PL from excitons [17, 44, 45]. However, the fabrication of metasurfaces is complicated and the integration of metasurfaces with monolayer TMDCs is difficult. In comparison, planar waveguide structures without any complex patterning process can also provide electric field enhancement used to modify the exciton dynamics in monolayer TMDCs. On the other hand, the emission from defect-bound excitons can be enhanced by exciting a monolayer TMDC with a large size attached on a planar waveguide structure. Since the self-absorption effect is much weaker for defect-bound excitons as compared with neutral excitions, the emission from defect-bound excitons can propagate a longer distance [46]. As result, the emission from defect-bound excitons is enhanced at the collection point because of its nonlocal feature. In a previous study, we showed that a dielectric-metal heterostructure composed of a Si_3_N_4_ layer and a thin Ag film can support transverse electric (TE) polarized waves localized on the surface of the Si_3_N_4_ layer. Owing to the enhancement in the in-plane electric field, strong coupling between the TE wave and the two excitons in a WS_2_ monolayer attached on the Si_3_N_4_/Ag heterostructure was successfully demonstrated [47, 48]. Therefore, it is interesting to investigate the exciton dynamics in monolayer TMDCs excited by the TE wave supported by a dielectric-metal heterostructure.

In this work, we convert the excitation laser light into the TE wave propagating on a Si_3_N_4_/Ag heterostructure and excite a WS_2_ monolayer attached on the heterostructure. In this way, we reveal simultaneously the emissions from neutral excitons (*X*_A_), charge excitons (trions) (*X*_T_), and defect-bound excitons (*X*_D_) in the PL spectrum of the WS_2_ monolayer at room temperature. We examine the dependences of the emission intensities from the three excitons on the excitation position and excitation intensity. Our findings suggest a new way to excite the defect-bound excitons in WS_2_ monolayer and investigate multi-exciton physics at room temperature.

## 2 Results and discussion

**Figure 1:**
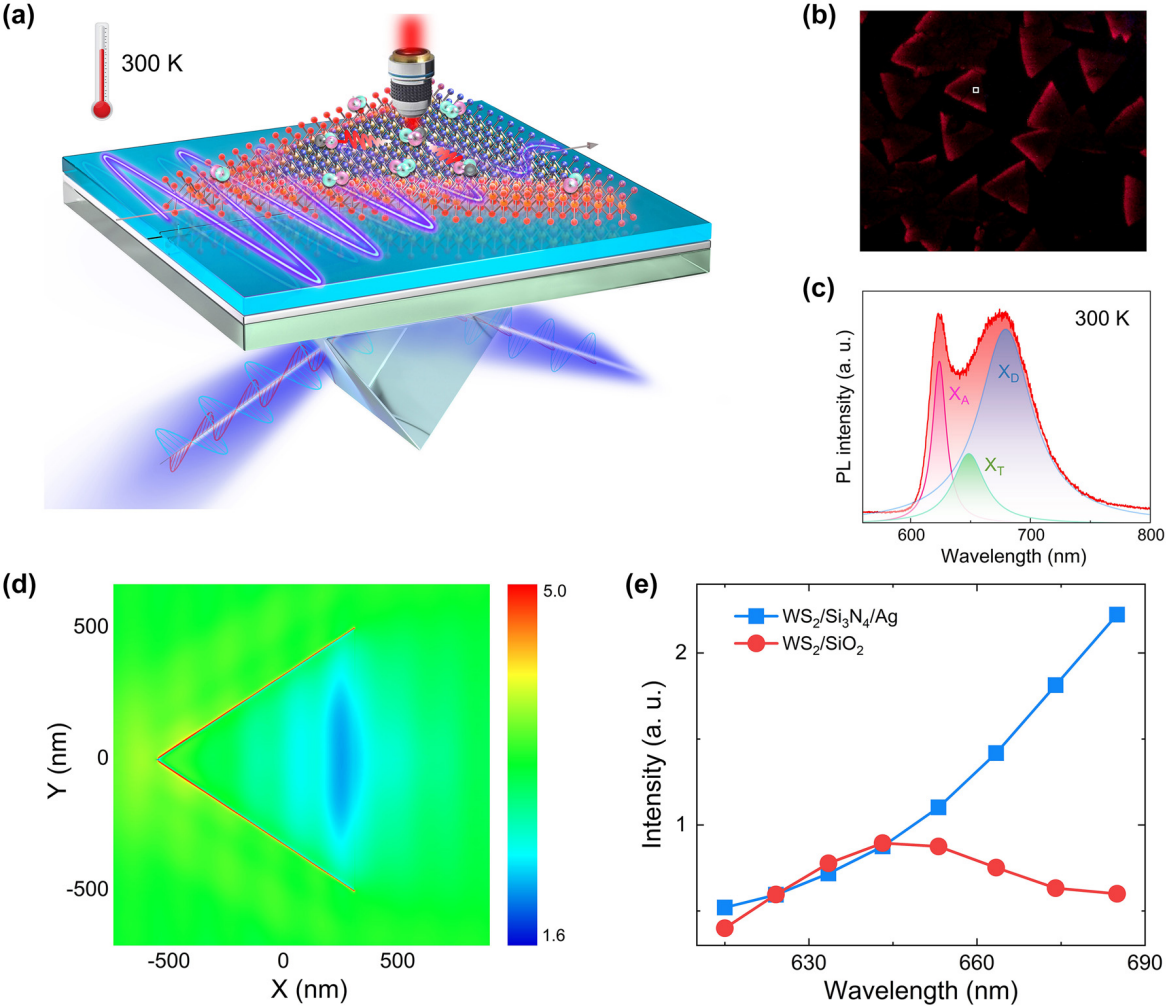
PL spectra of monolayer WS_2_ from TE wave excitation. (a) Schematic showing the excitation of a WS_2_ monolayer placed on a Si_3_N_4_/Ag heterostructure by coupling the laser light as a TE wave propagating on the heterostructure. The emissions from defect-bound excitons at different locations can be collected by an objective due to the existence of the waveguide structure. (b) Optical image of triangular WS_2_ monolayers placed on Si_3_N_4_/Ag heterostructure and excited by 405-nm laser light propagating as a TE wave. (c) Typical PL spectrum of a WS_2_ monolayers placed on the Si_3_N_4_/Ag heterostructure. Also shown are the fitting of the PL spectrum by Lorentz lineshapes. (d) In-plane electric field distribution (*E*_*xy*_) calculated for a WS_2_ monolayer placed on the Si_3_N_4_/Ag heterostructure. (e) Dependence of the radiation intensity on the wavelength calculated for a WS_2_ monolayer placed on the Si_3_N_4_/Ag heterostructure and a SiO_2_ substrate.

Monolayer WS_2_ exhibits a strong exciton resonance in the visible light spectrum. Thus, a laser beam with in-plane polarization will greatly boost the interaction with a WS_2_ monolayer, leading to enhanced PL intensity. In Figure 1a, we show schematically the interaction of a TE wave generated on the surface of a Si_3_N_4_/Ag heterostructure with a WS_2_ monolayer attached on the heterostructure. The heterostructure is composed of a 50-nm-thick Ag film deposited on a SiO_2_ substrate followed by a 100-nm-thick Si_3_N_4_ layer. Physically, the TE waves supported by the Si_3_N_4_/Ag heterostructure belong to substrate-modulated waveguide modes, similar to those described in previous studies [47, 48]. In this case, the whole WS_2_ monolayer can be excited by the TE wave localized on the surface of the Si_3_N_4_ layer [48] (see Figure S1 in the Supporting Information). It implies the generation of defect-bound excitons in all possible locations. In addition, the exciton dynamics in the WS_2_ monolayer will be modified by the Si_3_N_4_/Ag heterostructure. Finally, the emissions from defect-bound excitons at different locations can propagate to the collecting point with small attenuation, leading to an enhanced intensity in the PL spectrum (see Figure S2 in the Supporting Information). In Figure 1b, we show the optical images of triangular WS_2_ monolayers recorded by using a charge coupled device (CCD). In this case, WS_2_ monolayers are excited by 405-nm laser light coupled into the Si_3_N_4_/Ag heterostructure as a TE wave at an incident angle of *θ* = 80° (see Figure 1a). It is noticed that each WS_2_ monolayer emits red PL with enhanced intensity at left-side edges. In Figure 1c, we present a typical PL spectrum collected from a point on a WS_2_ monolayer by using an objective and analyzed by using a spectrometer at room temperature (see Figure 1a). It can be fitted by three Lorentz lineshapes, which corresponds to the emissions from neural excitons, charge excitons and defect-bound excitons respectively. The PL peak at ∼680 nm is attributed to the emission from defect-bound excitons. This assignment is confirmed by the experiment described in the following. Comparing with the conventional point excitation which exhibits only the emission from neutral excitons, the excitation of WS_2_ monolayer by using TE wave reported in this work provides a simple and effective way for revealing defect-bound excitons at room temperature, which is valuable for studying the multi-exciton physics in two-dimensional materials at room temperature and helpful for developing photonic devices based on monolayer TMDCs.

In Figure 1d, we show the in-plane electric field distribution calculated for a triangular WS_2_ monolayer placed on the Si_3_N_4_/Ag heterostructure and excited by the TE wave. The TE wave propagates on the surface of the Si_3_N_4_ layer from left to right. It is noticed that a strong localization of the electric field is achieved at the front edges of the WS_2_ monolayer, which is firstly excited by the TE wave. The enhancement factor is larger than that at the rear edge by a factor of ∼3.1. In order to see the influence of the Si_3_N_4_/Ag heterostructure on the emission properties of the excitons of the WS_2_ monolayer, we calculated the emission intensities of a horizontally oriented dipole source placed on the WS_2_/Si_3_N_4_/Ag heterostructure at different wavelengths. The result is shown in Figure 1e. The emission intensities of the same dipole source placed on a WS_2_/SiO_2_ substrate is also provided for comparison. It is found that the emission intensity of the dipole source at ∼680 nm is enhanced by a factor of ∼5.0 as compared with that at ∼615 nm for the WS_2_ monolayer placed on the Si_3_N_4_/Ag heterostructure. This behavior is not observed for the WS_2_ monolayer placed on the SiO_2_ substrate, implying that the exciton dynamics of the WS_2_ monolayer can be modified by using the Si_3_N_4_/Ag heterostructure. As a result, the radiative recombination rate of the defect-bound excitons is greatly enhanced as compared with that of neutral excitons.

**Figure 2:**
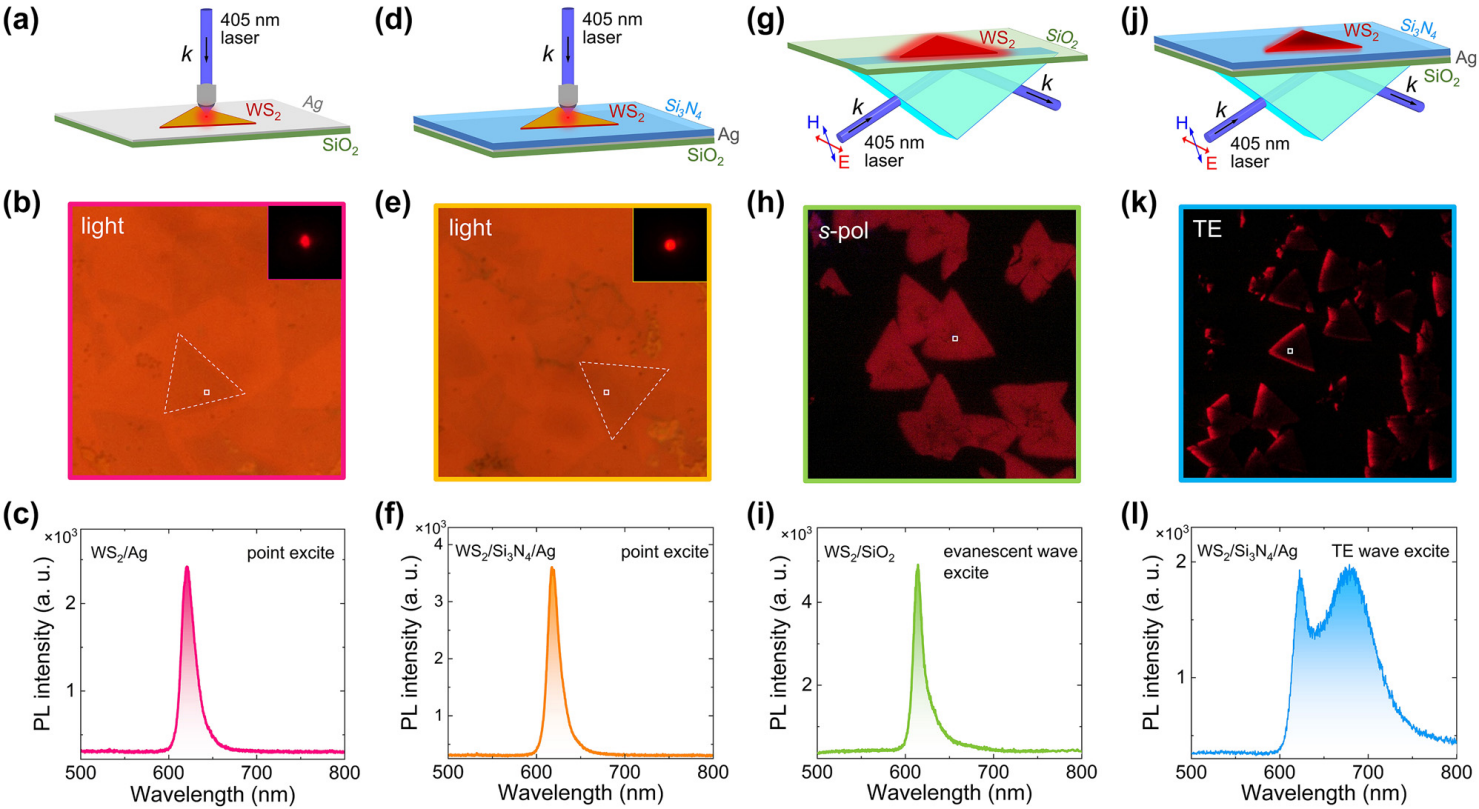
PL spectra of WS_2_ monolayer on different substrate. (a) Schematic showing the excitation of a WS_2_ monolayer placed on a Ag/SiO_2_ substrate by using a focused laser light. (b) Optical image of the WS_2_ monolayer (marked by a dashed triangle) placed on the Ag/SiO_2_ substrate. The CCD image of the PL from the WS_2_ monolayer is shown in the inset. (c) PL spectrum of the WS_2_ monolayer placed on the Ag/SiO_2_ substrate. (d) Schematic showing the excitation of a WS_2_ monolayer placed on a Si_3_N_4_/Ag heterostructure by using a focused laser light. (e) Optical image of the WS_2_ monolayer (marked by a dashed triangle) placed on the Si_3_N_4_/Ag heterostructure. The CCD image of the PL from the WS_2_ monolayer is shown in the inset. (f) PL spectrum of the WS_2_ monolayer placed on the Si_3_N_4_/Ag heterostructure. (g) Schematic showing the excitation of a WS_2_ monolayer placed on a SiO_2_ substrate by using the evanescent wave. (h) CCD image of triangular WS_2_ monolayers placed on the SiO_2_ substrate and excited by the evanescent wave. (i) PL spectrum of the WS_2_ monolayer placed on the SiO_2_ substrate and excited by the evanescent wave. (j) Schematic showing the excitation of a WS_2_ monolayer placed on a Si_3_N_4_/Ag heterostructure by laser light coupled into the heterostructure as a TE wave. (k) CCD image of triangular WS_2_ monolayers placed on the Si_3_N_4_/Ag heterostructure and excited by the TE wave. (l) PL spectrum of the WS_2_ monolayer placed on the Si_3_N_4_/Ag heterostructure and excited by the TE wave.

In order to understand the physical mechanisms responsible for the appearance of the defect-bound excitons in the PL spectrum of the WS_2_ monolayer placed on Si_3_N_4_/Ag heterostructure and excited by the TE wave, we compare the PL spectra of WS_2_ monolayers placed on different substrates and excited by different methods, as shown in Figure 2 (see Figure S3 in the Supporting Information). In Figure 2a–c, we show the PL measurement performed for a WS_2_ monolayer placed on an Ag/SiO_2_ substrate. The conventional point excitation and collection is employed in this case. Only a single peak (∼615 nm) originating from neutral excitons is observed [6, 9]. Similar result is found for the WS_2_ monolayer placed on a Si_3_N_4_/Ag heterostructure if the same excitation method is employed, as shown in Figure 2d–f (see Figure S4 in the Supporting Information). Although the exciton dynamics is expected to be modified by the Ag film and the Si_3_N_4_/Ag heterostructure, the emission from defect-bound excitons is still invisible in the PL spectrum. This behavior indicates that the excitation of the entire the WS_2_ monolayer instead of only a single point is crucial for revealing the emission from defect-bound excitons. For this reason, we also examined the PL spectrum of a WS_2_ monolayer placed on a SiO_2_ substrate and excited by using the evanescence wave, as shown in Figure 2g–i Unfortunately, the emission from defect-bound excitons is still very weak as compared with that of neutral excitons. It means that the modification in exciton dynamics is also necessary except the excitation of the entire WS_2_ monolayer. These requirements are fulfilled for the WS_2_ monolayer placed on Si_3_N_4_/Ag heterostructure, as shown in Figure 2j–l (see Figure S5 in the Supporting Information). In Figure 2l, one can clearly identify the emission from defect-bound excitons (∼680 nm) with intensity comparable to that from neutral excitons (∼615 nm) [6, 7, 9].

**Figure 3:**
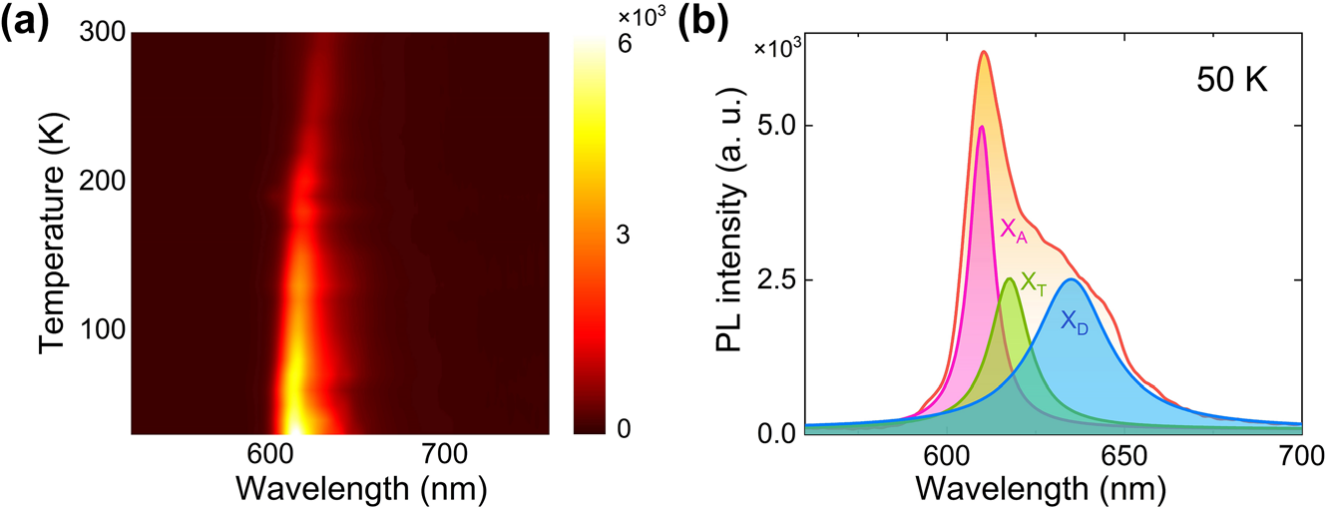
Low-temperature PL spectra of WS_2_ monolayer. (a) Temperature-dependent PL spectra of a WS_2_ monolayer placed on the Si_3_N_4_/Ag heterostructure and excited by a focused laser beam. (b) PL spectrum of the WS_2_ monolayer placed on the Si_3_N_4_/Ag heterostructure obtained at *T* = 50 K. The fitting of the PL spectrum by using Lorentz lineshapes is also provided.

In order to confirm that the broad PL peak at ∼680 nm originates from the emission of defect-bound exciton, we perform PL measurements for a WS_2_ monolayer at low temperatures, which is a conventional way to identify defect-bound excitons in monolayer TMDCs [6, 9]. In Figure 3a, we show the evolution of the PL spectrum of a WS_2_ monolayer with decreasing temperature. It is noticed that the PL intensity is increased while the PL bandwidth is broadened with decreasing temperature. In Figure 3b, we show the PL spectrum obtained at *T* = 50 K together with the fitting with Lorentz lineshapes. One can see clearly the contributions from neural, charge, and bound excitons, which are denoted as *X*_A_, *X*_T_ and *X*_D_, respectively. Although a blueshift of the PL peak is observed for all excitons at low temperatures, the similarity between the PL spectrum observed for the WS_2_ monolayer placed on the Si_3_N_4_/Ag heterostructure and excited by the TE wave and that obtained at a low temperature confirms the revealing of *X*_D_ at room temperature.

**Figure 4:**
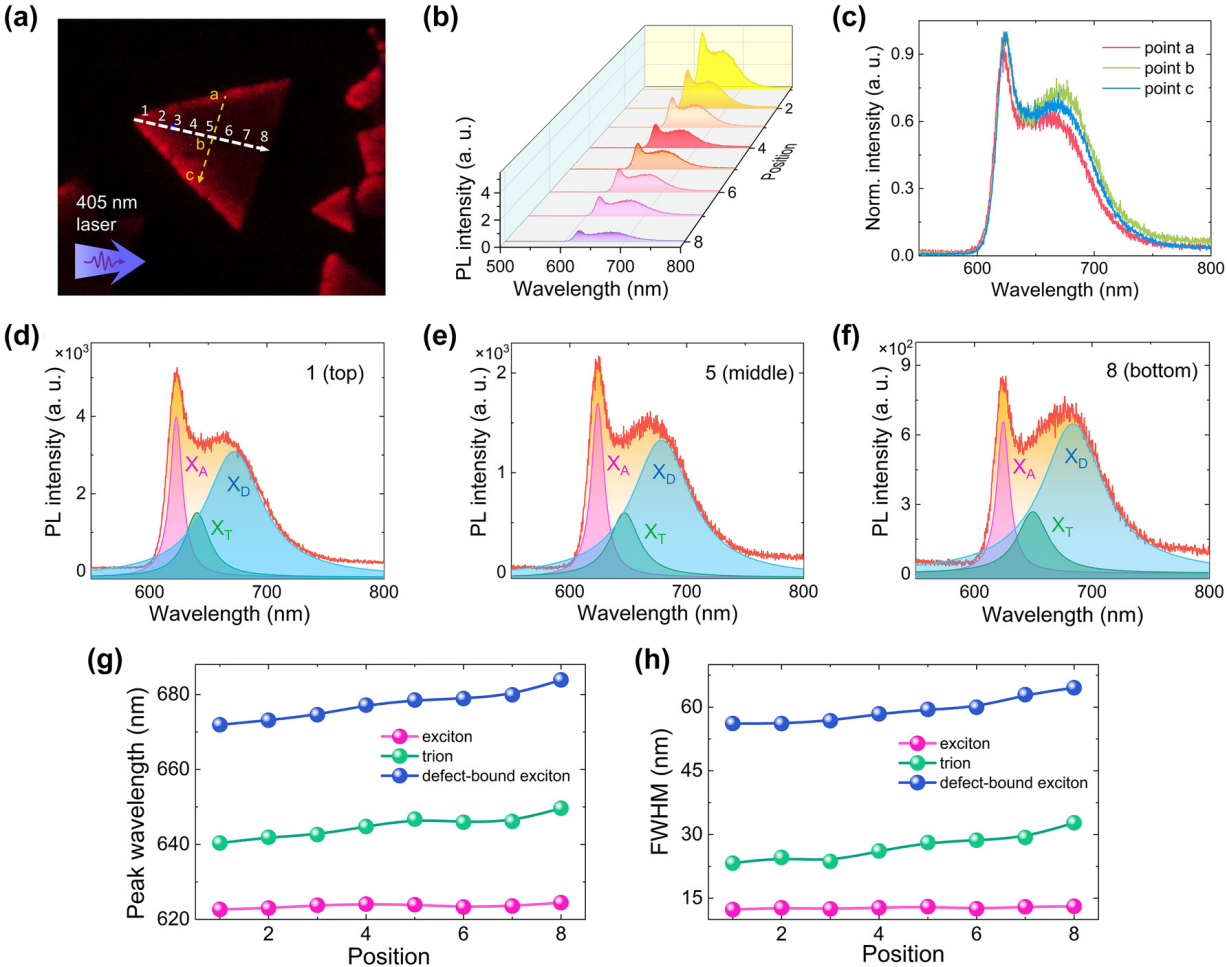
Efficiently exciting defect-bound excitons at different locations of the WS_2_ monolayer by TE wave. (a) CCD image of a triangular WS_2_ monolayer excited by 405-nm laser light coupled into the Si_3_N_4_/Ag heterostructure as a TE wave. The PL spectra are measured at different positions on the dashed line from the vertex (point 1) to the base (point 8) of the triangle and the dashed line parallel to the base of the triangle (point a-c). (b) PL spectra obtained at different positions (point 1–8) along the dashed line from the vertex to the base of the triangle. (c) The PL spectra obtained at different positions (point a–c) the dashed line parallel to the base of the triangle. (d–f) PL spectra measured at three different positions (point 1, 5, and 8) together with the fittings with multiple Lorentz lineshapes. (g) Position-dependent peak wavelength extracted for the three excitons (*X*_A_, *X*_T_, and *X*_D_) from the fitting of the PL spectrum. (h) Position-dependent bandwidth extracted for the three excitons (*X*_A_, *X*_T_, and *X*_D_) from the fitting of the PL spectrum.

Now we investigate in detail the emission from defect-bound excitons (*X*_D_) in the WS_2_ monolayer placed on the Si_3_N_4_/Ag heterostructure and excited by the TE wave. Since the PL intensity on the WS_2_ monolayer is not uniform, we first examine the PL spectra at different locations of the WS_2_ monolayer, as shown in Figure 4a. The PL spectra obtained at different positions along the two dashed lines are shown in Figure 4b and c. It is found that the overall PL intensity decreases from the vertex (point 1) to the base (point 8) of the triangular WS_2_ monolayer along the dashed line. However, it is noticed that the relative intensity of defect-bound excitons (*X*_D_) with respect to that of neutral excitons (*X*_A_) is increased. At point 8, the emission from *X*_D_ becomes comparable to that from *X*_A_. In comparison, no obvious change in the PL spectrum is observed along the other dashed line (from point a to point b), as shown in Figure 4c, implying that the emission from *X*_D_ is sensitive to the propagation direction of the TE wave. In Figure 4d–f, we show the PL spectra obtained at three positions (point 1, 5, and 8) together with the fittings with multiple Lorentz lineshapes, which correspond to the emissions from *X*_A_, *X*_T_, and *X*_D_, respectively. It can be seen that the contribution of *X*_D_ becomes more significant at the dark region (point 8). In order to gain a deep insight into the change in the PL spectrum, we extracted the peak wavelengths and bandwidths of the three excitons observed at different positions, as shown in Figure 4g and h. It is found that both the wavelength and bandwidth of *X*_A_ remain unchanged at different positions. In comparison, a redshift of the peak wavelength and a broadening of the bandwidth are observed for *X*_T_ and *X*_D_. The redshift and broadening appear to be more significant for *X*_D_, implying the emissions from *X*_D_ at different locations can be effectively collected at the base of the triangular WS_2_ monolayer, which is the latest region excited by the propagating TE wave. Actually, the emission from neutral excitons couples strongly to the TE waveguide mode because of their in-plane transition dipole moments. However, the strong self-absorption effect makes it decay fast during propagation. In comparison, the weak self-absorption for defect-bound excitons leads to a longer propagation distance, enhancing the emission from defect-bound excitons at the collection point. Thus, the nonlocal feature of defect-bound excitons makes it possible to reveal their emission in the PL spectrum of a WS_2_ monolayer at room temperature.

**Figure 5:**
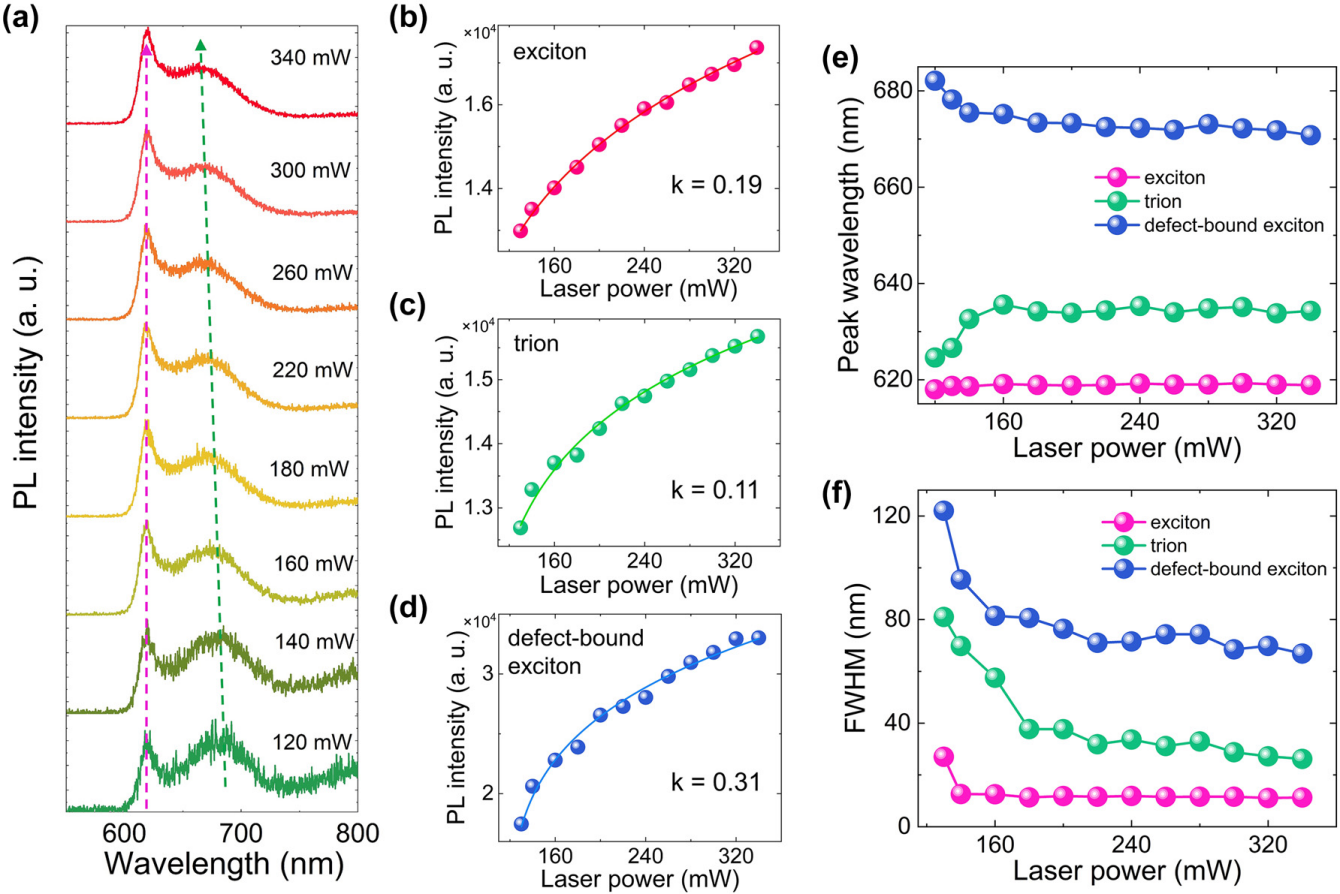
Manipulate the relative intensity of *X*_D_ by change the excitation condition. (a) PL spectra of a WS_2_ monolayer placed on the Si_3_N_4_/Ag heterostructure and excited by the TE wave with different laser powers. (b–d) Power-dependent integrated intensity extracted for *X*_A_, *X*_T_, and *X*_D_ from the fitting of the PL spectra obtained at different laser powers. (e) Dependence of the peak wavelength on the laser power extracted for *X*_A_, *X*_T_, and *X*_D_ from the fitting of the PL spectra obtained at different laser powers. (f) Dependence of the bandwidth on the laser power extracted for *X*_A_, *X*_T_, and *X*_D_ from the fitting of the PL spectra obtained at different laser powers.

So far, we have demonstrated that the emission from *X*_D_ can be revealed in the PL spectrum of a WS_2_ monolayer placed on a Si_3_N_4_/Ag heterostructure at room temperature by coupling the excitation laser light into the heterostructure as a TE wave. In practical applications, it is desirable that the relative intensity of *X*_D_ can be manipulated by simply changing the excitation condition. In Figure 5a, we show the PL spectra obtained at position 8 by using different laser powers (see Figure S6 in the Supporting Information). It is remarkable that relative intensities of *X*_A_, *X*_T_, and *X*_D_ can be modified by simply varying the laser power (*P*). Similarly, we can extract the integrated intensities (*I*) of *X*_A_, *X*_T_, and *X*_D_ at different laser powers by fitting the PL spectrum with multiple Lorentz lineshapes. The dependence of the integrated intensity on the laser power derived for *X*_A_, *X*_T_, and *X*_D_ are shown in Figure 5b–d, respectively. In all cases, the intensity increases rapidly at low laser powers (*P* < 180 mW) and slowly at high laser powers. Such a sublinear dependence on the laser power can be fitted by power law *I* ∝ *P* ^k^ [41, 49, 50]. The parameter k extracted for *X*_A_, *X*_T_, and *X*_D_ are ∼0.19, ∼0.11, and ∼0.31, respectively. It is noticed that the slopes observed in the dependence of the emission intensity on the excitation power for all excitons are smaller than those observed in the conventional point excitation. Moreover, the slope observed for defect-bound excitons is even larger than that observed for neutral excitons. The rapid attenuation of neutral excitons induced by the strong self-absorption effect as compared with defect-bound excitons is responsible for this abnormal behavior. The complex fitting results indicate the nonuniform excitation of the WS_2_ monolayer by the TE wave, implying the existence of multi-exciton channels in the emission (see Figure S7 in the Supporting Information). In Figure 5a, it is noticed that the peak wavelength of *X*_D_ is blue-shifted with increasing the laser power. In contrast, the peak wavelength of *X*_A_ peak remains almost unchanged. The blueshift is mainly caused by the band filling effect of defect states with increasing the excitation intensity [41, 51]. In Figure 5e and f, we present the dependences of the peak wavelength and bandwidth on the laser power extracted for the three types of excitons. While a blueshift is observed for the peak wavelength of *X*_D_, the peak wavelength of *X*_T_ exhibits a redshift with increasing laser power. The peak wavelength of *X*_A_ remains nearly unchanged. In all cases, one can see a narrowing of the bandwidth with increasing laser power, particularly in the emissions of *X*_T_ and *X*_D_. Peak has the opposite change in the center wavelength with increasing the power. Both the change in the peak wavelength and bandwidth induced by the laser power indicate that the binding energy of electrons and holes can be renormalized by the excitation laser light, which is beneficial to the formation of defects by trapping oxygen. As a result, the emission from defect-bound excitons can be visualized even at room temperature. This process is similar to the introduction of defects in WS_2_ monolayer by using high-energy electron beam, revealing the emission of *X*_D_ at room temperature [52] (see Figure S8 in the Supporting Information). In this case, however, the structure of the WS_2_ monolayer is modified, limiting it practical applications.

## 3 Conclusions

In conclusion, we investigated the PL spectrum of a WS_2_ monolayer placed on a Si_3_N_4_/Ag heterostructure and excited by coupling 405-nm laser light into the heterostructure as a TE wave. By utilising the TE wave to excite the entire WS_2_ monolayer, modifying the exciton dynamics through the heterostructure, and enabling emission propagation along the waveguide structure, we successfully revealed the emission from defect-bound excitons at room temperature. We observed that the intensity of the defect-bound excitons strongly depends on the position along the propagating direction of the excitation TE wave and can be comparable to that of other excitons. Furthermore, we demonstrated that the relative intensity, peak wavelength, and bandwidth of the defect-bound excitons can be manipulated by simply adjusting the excitation laser power. Our findings highlight the advantages of dielectric-metal heterostructures in exciting defect-bound excitons, which are valuable for studying the multi-exciton physics in two-dimensional materials at room temperature and hold significance for the development of photonic devices based on monolayer TMDCs.

## 4 Methods

### 4.1 Sample preparation and characterization

In this work, the WS_2_/Si_3_N_4_/Ag heterostructure was fabricated by using the method reported previously [48]. First, a 50-nm-thick Ag film was deposited on a silica (SiO_2_) substrate by using electron beam evaposition. Then, a 100-nm-thick Si_3_N_4_ layer was deposited on the Ag film via high-frequency plasma-enhanced chemical vapor deposition (HF-PECVD). Finally, triangular WS_2_ monolayers grown by the CVD method were transferred onto the surface of the Si_3_N_4_/Ag heterostructure. The morphologies of WS_2_ monolayers were examined by SEM observation (Gemini 500, Zeiss). The density of defects was controlled by adjusting the irradiation time of the high-energy electron beam.

### 4.2 Optical characterization

The optical images and the PL spectra of WS_2_ monolayer placed on different substrates were measured by using an inverted microscope (Axio Observer A1, Zeiss) equipped with a spectrometer (SR-500i-B1, Andor) and a color charge coupled device (CCD) (DS-Ri2, Nikon). Two methods were employed to excite the WS_2_ monolayers placed on the Si_3_N_4_/Ag heterostructure. In the conventional excitation method, a 405-nm laser beam was introduced into the microscope and focused on WS_2_ monolayers by using a 100× objective. In the excitation by using the TE wave, the 405-nm laser beam was coupled into the Si_3_N_4_/Ag heterostructure as a TE wave by using a prism, as schematically shown in Figure 1a. In both cases, the PL from the WS_2_ monolayer was collected by using a 100× objective and directed to the spectrometer for analysis. The temperature-dependent PL spectra were measured using an Ultra-low vibration closed cycle cryostat (DE204PF-DMX-20-OM, ARS).

### 4.3 Numerical simulation

The numerical simulations were performed by using the finite-difference time-domain (FDTD) method (FDTD solution, https://www.lumerical.com). In the numerical simulation, the refractive index of Si_3_N_4_ was based on the measured data. The dielectric constants of Ag and WS_2_ monolayer were taken from the previous literature [53, 54]. The thickness of the WS_2_ monolayer was set to be 1.0 nm. The refractive index of the surrounding media was chosen to be 1.0. The dipole source was placed inside the WS_2_ monolayer to calculate the radiation intensity of the WS_2_/Si_3_N_4_/Ag heterostructure. The *p*- and *s*-polarized plane waves were used to calculate the electric and magnetic field distributions of TM and TE waves supported by Ag film and Si_3_N_4_/Ag heterostructures, respectively. The smallest mesh size as small as 0.5 nm was used to obtain converged simulation results, and perfectly matched layer boundary condition was employed to terminate the finite simulation.

## Supplementary Material

Supplementary Material Details
