# Supplementary material for: Revealing defect-bound excitons in WS2 monolayer at room temperature by exploiting the transverse electric polarized wave supported by a Si3N4/Ag heterostructure
Source: Nanophotonics. 2023 Nov 22;12(24):4485–94. doi: 10.1515/nanoph-2023-0560 (PMC11501904; doi:10.1515/nanoph-2023-0560)
Supplement: Supplementary file 1 — Supplementary Material Details [file j_nanoph-2023-0560_suppl_001.pdf]

Supporting Information

Revealing defect-bound excitons in WS_2 monolayer at room temperature by exploiting the transverse electric polarized wave supported by a Si_3N_4/Ag heterostructure

Shulei Li[1,*], Fu Deng[3,*], Lidan Zhou[4], Zhenxu Lin[2], Mingcheng Panmai[2], Shimei Liu[2], Yuheng Mao[2], Jinshan Luo[2], Jin Xiang[5], Jun Dai[1], Yunbao Zheng[1], and Sheng Lan[2,*]

[1] *School of Optoelectronic Engineering, Guangdong Polytechnic Normal University, Guangzhou 510665, China*

[2] *Guangdong Provincial Key Laboratory of Nanophotonic Functional Materials and Devices, School of Information and Optoelectronic Science and Engineering, South China Normal University, Guangzhou 510006, China*

[3] *Department of Physics, Hong Kong University of Science and Technology, Kowloon, Hong Kong, China, Hong Kong 999077, China*

[4] *State Key Laboratory of Optoelectronic Materials and Technologies, School of Electronics and Information Technology, Sun Yat-sen University, Guangzhou 51006, China*

[5] *Key Laboratory of Optoelectronic Technology and Systems (Chongqing University), Ministry of Education, School of Optoelectronic Engineering, Chongqing University, Chongqing 400044, China*

[*] Correspondence: slan@scnu.edu.cn (S. Lan); shuleili@gpnu.edu.cn (S. Li); fu_deng@foxmail.com (F. Deng)

Supplementary Note1: Electric and magnetic field distribution for the TM wave and TE wave

We compare the electric and magnetic field distributions of TM waves (i.e., the SPPs) and TE waves. SPPs is a TM wave propagating on the surface of a metal can only be excited by using p-polarized light, which have a strong localization of electric field. However, SPPs have large damping rates result in the broad linewidths, as shown in Figure S1a. In Figure S1b,c, we show the in-plane electric component (Ex) and magnetic components (Hy) for the TM waves at the incidence angle of $\theta = 45.5°$, which can be seen the electric and magnetic fields are mainly distributed in the air. From previous studies, TE waves supported by the Si3N4/Ag heterostructure belong to substrate-modulated waveguide modes, as shown in Figure S2d. We found that TE waves are spatially separated, leading to higher quality factors or narrow linewidths, as shown in Figure S2e,f. More importantly, compared with the corresponding TM waves, the in-plane electric field of TE waves can be perfectly coupled with excitons in WS_2 monolayers, the enhancement factor is larger than that obtained in the TM wave by a factor of ~3.0.

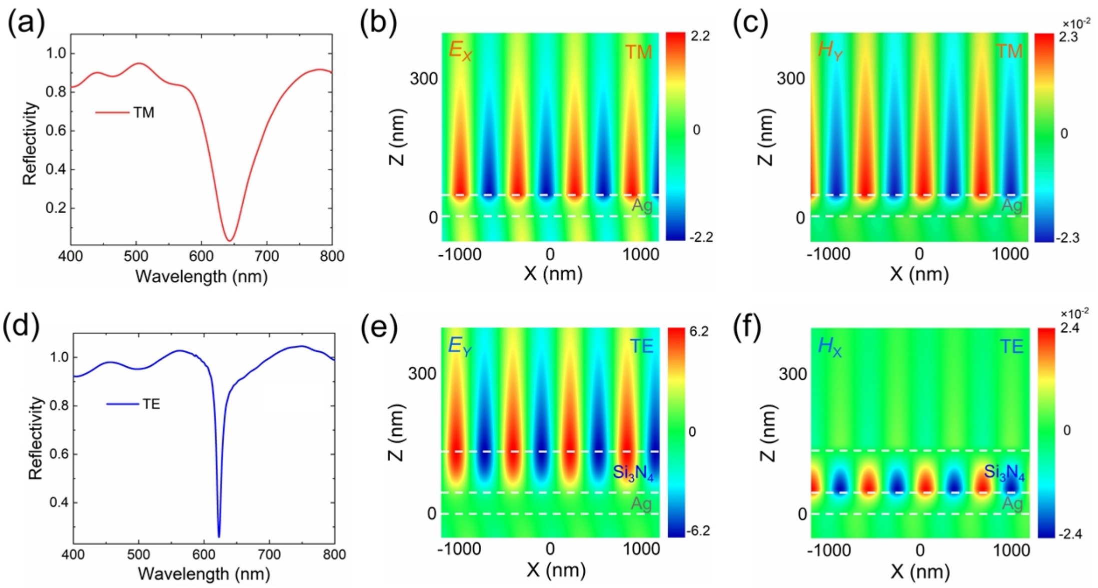

Figure S1: (a) Simulated reflectivity spectra for the Ag film by using the TM wave generated with p-polarized white light at 45.5° incidence angles. (b,c) The *Ex* and *Hy* components for the TM wave. (d) Simulated reflectivity spectra for the Si_3N_4/Ag heterostructure by using the TE wave generated with s-polarized white light at 50° incidence angles. (e,f) The *Ey* and *Hx* components for the TE wave.

Supplementary Note 2: Radiation intensity of WS_2 on different substrates

We calculated the emission intensities of TE wave propagating in WS_2 monolayer placed on the Si_3N_4/Ag heterostructure by using horizontally oriented dipole sources. We placed three point monitors, one above the dipole sources, one at 2.5 μm, and one at 5 μm, as shown in the inset of Figure S2. The emission intensities of the different points were provided for comparison, as shown in Figure S2. We found that although the emission intensity gradually decreases with the increase of the propagation distance, the emission intensity of the dipole source at ~680 nm is significantly enhanced compared with that at ~615 nm for the WS_2 monolayer. Therefore, the emissions from defect-bound excitons at different locations can propagate to the collecting point.

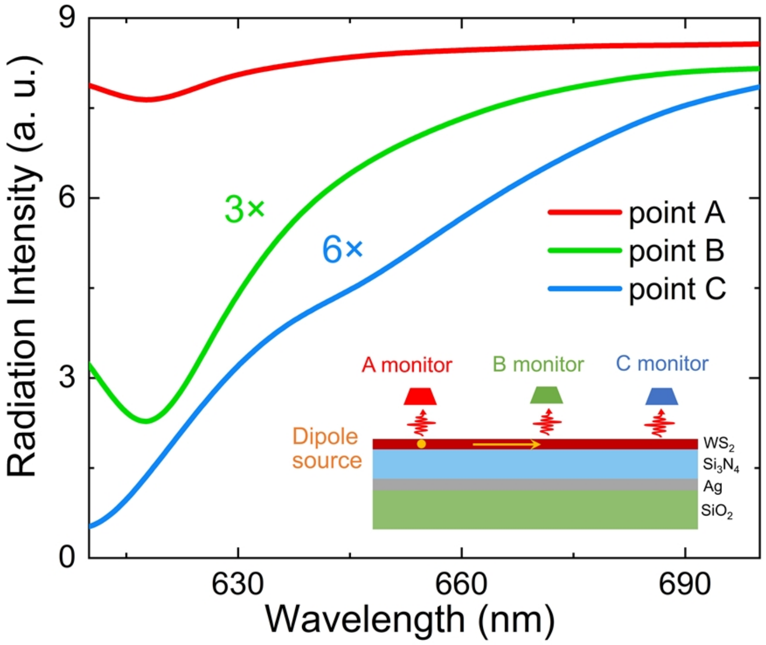

Figure S2: Radiation intensity at different positions on the WS_2/Si_3N_4/Ag heterostructure

Supplementary Note 3: Excitation methods of the WS_2 monolayer placed on different substrates

Generally, the excitation of the WS_2 monolayer is normally performed by introducing the laser beam into microscopy and focusing on WS_2 monolayers by using an objective, so-called conventional point excitation and collection methods, as schematically shown in Figure S3a. We know that the excitation of the entire the WS_2 monolayer instead of only a single point is crucial for revealing the emission from defect-bound excitons. For this reason, we used two methods that can excite in-plane electric field via the so-called Kretschmann-Raether configuration by using *s*-polarized light. In Figure S3b, we show schematically the interaction of an evanescence wave generated on the surface of a SiO_2 substrate with a WS_2 monolayer. In Figure S3c, we show schematically the interaction of a transverse electric (TE) wave by laser light coupled into the Si_3N_4/Ag heterostructure with a WS_2 monolayer attached on the heterostructure.

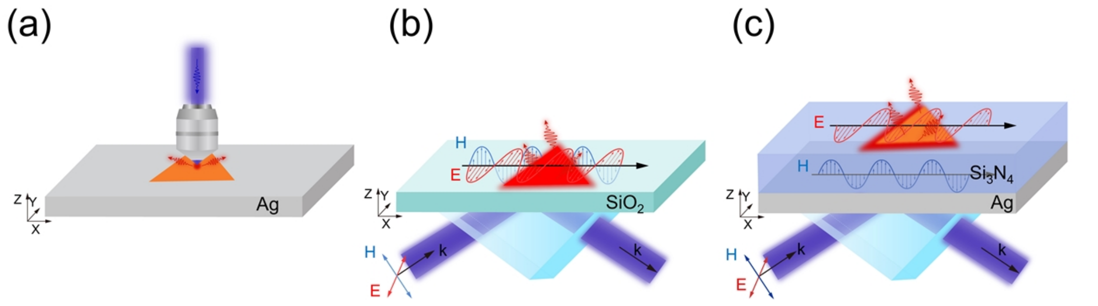

Figure S3: (a) Schematic showing the excitation of a WS_2 monolayer placed on a Ag/SiO_2 substrate by using a focused laser light. (b) Schematic showing the excitation of a WS_2 monolayer placed on a SiO_2 substrate by using the evanescent wave. (c) Schematic showing the excitation of a WS_2 monolayer placed on a Si_3N_4/Ag heterostructure by laser light coupled into the heterostructure as a TE wave.

Supplementary Note 4: Excitation of monolayer WS_2 on different substrates

We compared the PL spectra of WS_2 on the surface of SiO_2 substrate and Si_3N_4/Ag heterostructures by using a focused laser light at room temperature, as shown in Figure S4. There are a typical PL spectra caused by the X_A, the emission from defect-bound excitons is still invisible in the PL spectrum. A similar result is found for the WS_2 monolayer placed on the Ag structure.

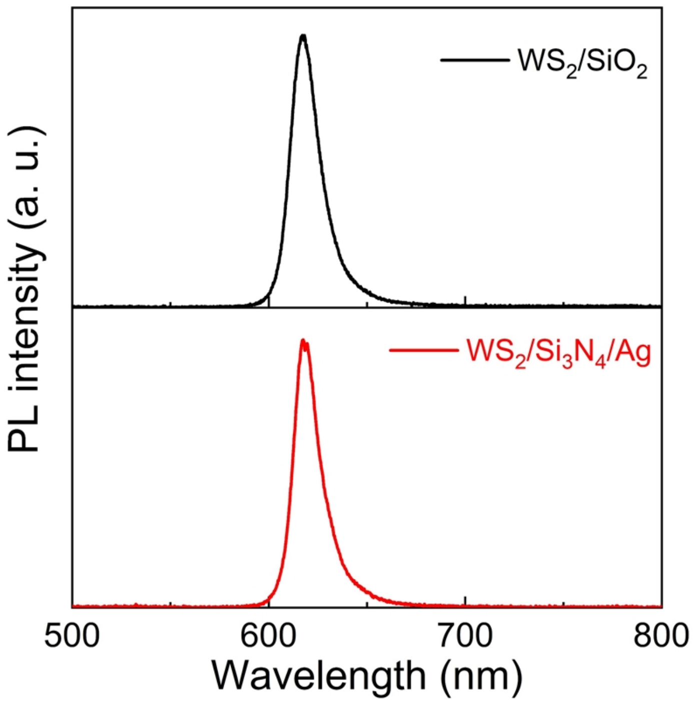

Figure S4: PL spectrum of the WS_2 monolayer placed on the SiO_2 substrate and the Si_3N_4/Ag heterostructures

Supplementary Note 5: Manipulation excite-trion coupling in a $WS_2/Si_3N_4/Ag$ heterostructure

It is mentioned that the TE wave has the in-plane electric field localized on the top surface of the Si_3N_4/Ag heterostructure. For this reason, the WS_2 monolayer has a stronger interaction with TE waves generated by coupling a 405 nm laser light into the Si_3N_4/Ag heterostructure. In Figure S5, we experimentally measured PL spectra for the WS_2 monolayer placed on the Si_3N_4/Ag heterostructure and excited using a 405 nm laser TE laser beam at different incidence angles. It is noticed that the typical asymmetric PL spectra at high laser powers are due to the creation of charge excitons at an incident angle of $\theta = 80°$, which are decomposed into the contributions of neural excitons and charge excitons. It was observed that the PL from the WS_2 monolayer becomes dominated by the emission from charge excitons by simply varying the incidence angle from 80° to 81.2°.

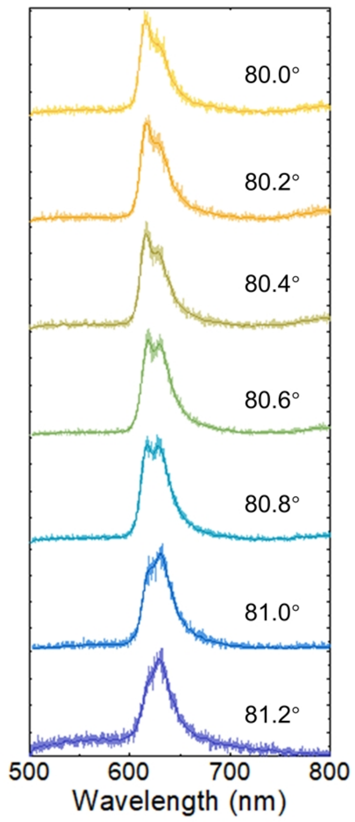

Figure S5: PL spectra measured for a WS_2 monolayer placed on the Si_3N_4/Ag heterostructure at different incidence angles.

Supplementary Note 6: PL spectra of the edge positions of WS_2 monolayer placed on Si_3N_4/Ag heterostructures by changing different laser power

We measured the PL spectrum of the edge positions of a WS_2 monolayer placed on the Si_3N_4/Ag heterostructures at room temperature by coupling the 405 nm-laser light into the heterostructure as a TE wave, as shown in Figure S6a,b. It was noticed that the relative intensity of X_D can be manipulated by simply changing the excitation power. We find the same result as at position 8, with the increase of the laser power, the peak wavelength of X_D is blue-shifted and the peak wavelength of X_A peak remains almost unchanged.

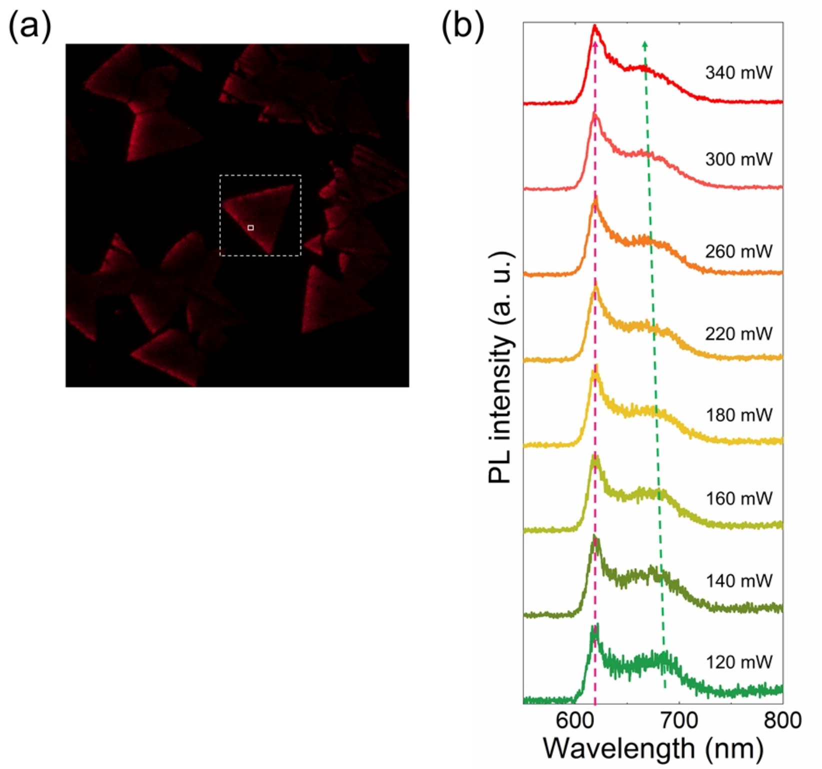

Figure S6: (a) CCD image of a triangular WS_2 monolayer excited by 405-nm laser light coupled into the Si_3N_4/Ag heterostructure as a TE wave. (b) PL spectra of a WS_2 monolayer placed on the Si_3N_4/Ag heterostructure and excited by the TE wave with different laser powers.

Supplementary Note 7: Power-dependent intensity for neutral excitons (X_A) of a WS_2 monolayer placed on a SiO_2 substrate with different laser powers

The dependence of the intensity on the laser power for neutral excitons (X_A) of a WS_2 monolayer placed on a SiO_2 substrate using a focused laser light is shown in Figure S7. It is found that the relationship between PL intensity (I) and excitation power (P) follows the power law $I \propto P^k$, where k is ~1.1. As a general result it has been found that $1< k <2$ for exciton transition.

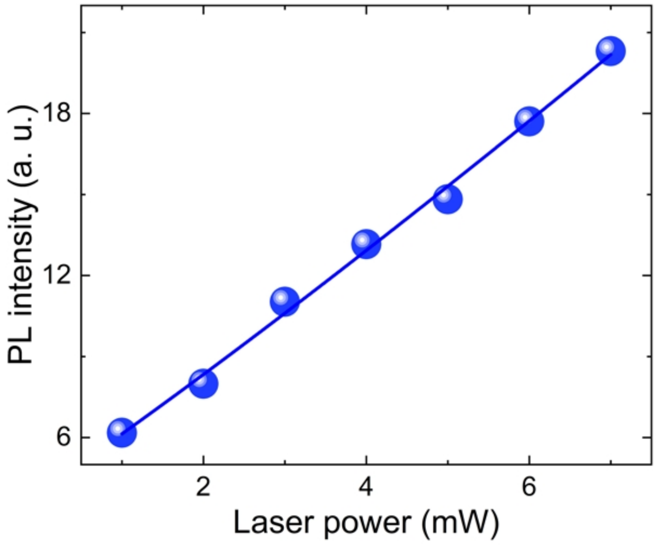

Figure S7 Power-dependent intensity for X_A from the PL spectra obtained at different laser powers.

Supplementary Note 8: The dependence of the PL intensity for a WS_2 monolayer placed on a SiO_2 substrate

It has been reported that precisely controlling the energy of the electron beam can introduce defects in the two-dimensional materials, achieving optical properties modulation of the material. Thus, We used a high-energy electron beam to irradiate a WS_2 monolayer placed on the SiO_2 substrate and investigated the emission from X_D in the WS_2 monolayer at room temperature. In Figure S8a, we show the optical images of triangular WS_2 monolayers by using 405nm laser light after irradiation with a high-energy electron beam. The white dashed line represents the irradiated area. The area with overlapping white dashed lines that the area has been irradiated multiple times. It is found that the relative intensity of X_D with respect to that of X_A is increased at different positions on the blue dashed line from point 1 to point 5. As the irradiation time increases, further confirming the trend of X_D increasing with electron beam irradiation time, indicating that the electron beam has disrupted the structure of a WS_2 monolayer, increasing the number of X_D, as shown in Figure S8b.

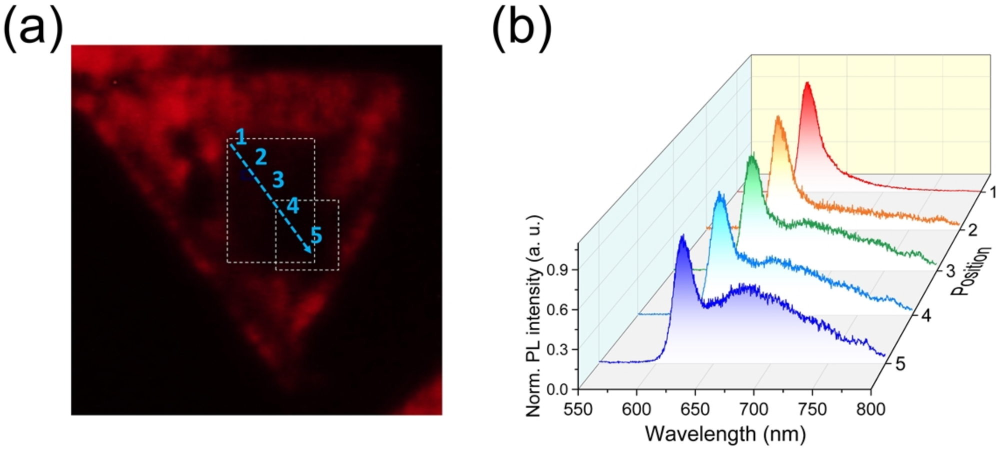

Figure S8: (a) CCD image of a triangular WS_2 monolayer after the high-energy electron beam irradiation. The PL spectra are measured at different positions on the blue dashed line from point 1 to point 5. (b) PL spectra obtained at different positions (point 1-5) along the blue dashed line.
